# Body fat percentage and CRP correlates with a composite score of vascular risk markers in healthy, young adults - The Lifestyle, Biomarkers, and Atherosclerosis (LBA) study

**DOI:** 10.1186/s12872-020-01376-6

**Published:** 2020-02-11

**Authors:** Paul Pettersson-Pablo, Yang Cao, Torbjörn Bäckström, Torbjörn K. Nilsson, Anita Hurtig-Wennlöf

**Affiliations:** 1 Department of Laboratory Medicine, Faculty of Medicine and Health, Örebro University Hospital, Örebro, Sweden; 2 School of Medicine, Faculty of Medicine and Health, Örebro University, Örebro, Sweden; 3 Department of Medical Biosciences/Clinical Chemistry, Umeå University, Umeå, Sweden; 4 Clinical Epidemiology and Biostatistics, School of Medical Sciences, Örebro University, Örebro, Sweden; 5 Department of Clinical Science, Obstetrics and Gynecology, Umeå University, Umeå, Sweden; 6 School of Health, Faculty of Medicine and Health, Örebro University, Örebro, Sweden

**Keywords:** Cluster analysis, Cardiovascular risk, Endothelial dysfunction, Obesity, Atherosclerosis, Young adults, Body fat percentage, CRP

## Abstract

**Background:**

Identification of early signs of atherosclerosis in young adults have the potential to guide early interventions to prevent later cardiovascular disease. We therefore analyzed measures of vascular structure and function and biomarkers of cardiovascular risk in a sample of young healthy adults.

**Methods:**

Pulse-wave velocity (PWV), carotid-intima media thickness (cIMT) and augmentation index (AIX) were measured in 834 healthy non-smokers (ages 18.0–25.9). Emphasis was put on discriminating between individuals having a vascular structure and function associated with a higher or lower risk, and cluster analysis algorithms were employed to assign the subjects into groups based on these vascular measurements. In addition, a vascular status score (VSS) was calculated by summarizing the results according to quintiles of the vascular measurements. The associations between VSS and cardiovascular biomarkers were examined by regression analyses.

**Results:**

The cluster analyses did not yield sufficiently distinct clustering (groups of individuals that could be categorized unequivocally as having either a vascular structure and function associated with a higher or lower CVD risk). VSS proved a better classificatory variable. The associations between VSS and biomarkers of cardiovascular risk were analyzed by univariable and multivariable regressions. Only body fat percentage and C-reactive protein (CRP) were independently associated with VSS.

**Conclusions:**

A VSS calculation, which integrates PWV, cIMT, and AIX measurements is better suited for cardiovascular risk evaluation in young adults than cluster analyses. The independent associations of VSS with body fat percentage and CRP highlight the decisive role of adiposity and systemic inflammation in early atherosclerotic progression and suggests a subordinate role of insulin and lipid metabolism in this age span.

## Background

The progression from a silent presence of atherosclerosis to symptomatic disease is characterized by a gradual buildup of plaques that are initially asymptomatic [1, 2]. Autopsies of children have shown that the atherosclerotic process begins early [3, 4]. The process typically evolves over half a lifetime and cardiovascular disease (CVD) is accordingly uncommon in young individuals [5, 6], with so called premature CVD representing only a minor part of total cardiovascular incidents. The long duration of this preclinical phase stresses the importance of studying the early stages of vascular pathology [7]. Risk factors present in childhood predict a higher lifetime risk of CVD [8, 9], suggesting that cardiovascular morbidity and mortality might be counteracted by early detection followed by intervention in individuals at risk.

Physiological measurements of vascular stiffness and vessel wall thickness serve as surrogate risk markers of future cardiovascular risk [10, 11]. Well-established and widely used vascular measures are carotid intima-media thickness (cIMT), pulse wave velocity (PWV) and augmentation index (AIX) [12–14]. They measure different vascular properties in distinct parts of the arterial tree, but are not entirely independent of each other, and covary to some extent [15]. An individual with increases in markers of both stiffness and thickness would therefore be thought to have a more pronounced preclinical atherosclerosis than an individual with only a single increased marker.

In female subjects, contraceptive use was included in the model to examine its interaction with vascular structure and function. Studies examining the effect of exogenously administered estrogen on vascular measurements have yielded conflicting results [16–19], and have not examined young populations. Exogenously administered estrogen is previously known to affect baseline levels of markers of systemic inflammation [20–22].

The purpose of the observational Lifestyle, Biomarkers and Atherosclerosis study (the LBA study) is to identify risk factors and biomarkers related to the early stages of the atherosclerotic process, and to make use of the findings to improve the preventive management of cardiovascular disease. In this study, we employed cluster analyses, mathematical pattern recognition methods used to identify subgroups within a given data set [23]. This was done in order to estimate cardiovascular risk in the different clusters, by comparing them with a set of established CVD biomarkers, encompassing metabolic markers, markers of inflammation and measurements of body composition [24–26].

## Methods

### Study population

The LBA sample consists of 834 Swedish adults between 18 and 25.9 years of age. Subjects were recruited by advertising at the Örebro University, and a local newspaper. A validated computerized questionnaire was used to assess the subjects’ lifestyle habits and served as verification that they met the inclusion criteria of being nonsmokers not suffering from chronic diseases [27]. The subjects were asked to report any medication they were using, including contraceptives. Based on the responses, females were grouped into estrogen containing contraceptive users (hereafter called “estrogen users”) and non-estrogen containing contraceptive users (“non-estrogen users”). A small portion of the subjects did not report the name of the contraceptive, but only that they were using some kind of oral contraceptives. In Sweden, combined estrogen and gestagen containing contraceptives are the most common choice of oral contraceptives [28]. Therefore, we made the decision to include these subjects in the estrogen users group. As a precautionary measure, the statistical analyses involving contraceptive use were recalculated, with the individuals who did not report the name of their contraceptive assigned to the non-estrogen group instead of the estrogen one.

### Body composition examination

Height was measured with a fixed stadiometer to the nearest 0.5 cm, with the subjects standing without shoes, heels together, back straight, and arms extended alongside the body. Body fat percentage was measured using a bioelectrical impedance body composition analyzer (Tanita BC-418 MA; Tanita Europe B.V., Amsterdam, the Netherlands). Adjustments were made with 1 kg for clothes and the standard setting was used.

### Vascular examinations

Blood pressure was measured after 15 min of rest in the left arm using a digital automated device (Dinamap V100; GE Healthcare, Buckinghamshire, UK) with Dura-Cuf (GE Critikon Dura-cuf; GE Medical Systems, Milwaukee, WI, USA). The subjects came for two visits on two different days to the university examination room and their blood pressure was registered on both occasions.

The cIMT was measured using a high-resolution ultrasound B-mode system, (GE Healthcare, Vivid E9, Chicago, Illinois, US) with a 12 MHz linear array transducer, as previously described [29]. An average of three measurements was reported for each subject [30].

Stiffness measures (PWV and AIX) were registered using applanation tonometry, using SphygmoCor (AtCor Medical Pty Ltd., SphygmoCor, Sydney, Australia) as previously described [31–33].

PWV was measured in the supine position. Carotid and femoral pulse waves were recorded with simultaneously ECG recording, and the PWV (m/s) calculated as PWV = distance between measurement locations (m) / transit time (s) for the pulse wave. A higher PWV indicates an increased vessel stiffness.

For AIX, the radial artery tonometry was performed at the subject’s right wrist. The aortic pressure waveform was derived from the radial waveform by a validated transfer function. AIX is calculated from the aortic pressure waveform and adjusted to heart rate 75 beats per minute (AIx_HR75). An average of AIx_HR75 from three measurements was reported for each subject. A higher AIX indicates an increased vessel stiffness [34].

### Serum biomarker analyses

Samples were collected after an overnight fast into sodium citrate fluoride vacutainer tubes for glucose analysis and serum and plasma vacutainer tubes for the rest of the analyses (BD Vacutainer; BD AB, Stockholm, Sweden). Serum was left to clot for at least 30 min before centrifugation and subsequent analysis. CRP, Orosomucoid, Apolipoprotein A-1 (Apo A-1) and Apolipoprotein B (Apo B) were analyzed on a Siemens ADVIA 1800 Chemistry instrument with a coefficient of variation (CV) of 5% at 0.74 mg/L with the Siemens High Sensitivity CRP Assay (ADVIA 1800 Chemistry System; Upplands Väsby, Sweden). The Apo A-1 assay had a CV of 4% at 0.9 g/L and the Apo B assay a CV of 5% at 1.5 g/L. Orosomucoid had a CV of 4% at 0.47 g/L using the DAKO orosomucoid immunoturbidimetry assay (Agilent, Santa Clara, California, USA). Total cholesterol (CHOL), Triglycerides (TG), high-density lipoprotein (HDL) and glucose were assayed colorimetrically with Vitros MicroSlide technology (5.1TM FS; Clinical Chemistry Instruments, Raritan, NJ, USA). Direct low-density lipoprotein (LDL) was assayed by a two-step colorimetric assay with Vitros MicroWell technology. CHOL (3% CV at 3.9 mmol/L), TG (CV of 4% at 1.3 g/L), HDL (6% CV at 1.0 mmol/L), LDL (5% CV at 2.4 mmol/L) and glucose (4% CV at 4.6 mmol/L) were analyzed on a Vitros 5.1 system (Vitros 5.1TM FS, Clinical Chemistry Instruments, Raritan, NJ, USA). Insulin was analyzed with the Abbott Architect Insulin Assay, a sandwich immunoassay using chemiluminescence detection with a CV of 7% at 8.0 mIU/L on an Architect i2000SR unit (Abbott, Abbot Park, IL, USA).

### Statistical analyses

Statistical analyses were performed with R ver 3.4.3 (R Foundation for Statistical Computing, Vienna, Austria). The vascular measures used in the cluster analyses were PWV, AIX and cIMT. The following cluster analysis algorithms were used: k means clustering, agglomerative nesting hierarchical cluster analysis (AGNES) and partitioning around medoids cluster analysis (PAM). Some of the algorithms allow machine identification of the ideal number of clusters in a model, while others require a trial and error approach, where each analysis is done with a predetermined number of clusters. Silhouette analyses permit the creation of silhouette plots visualizing the separation distance of the clusters, serving as an assessment of the quality of the cluster analysis models based on the number of predetermined clusters. A high silhouette index is suggestive of a good fit of the produced clusters. The optimal number of clusters was also evaluated using the Elbow method where a bend in the plot of the total within-cluster sum of squares is generally considered an indicator of the appropriate number of clusters.

In addition to the cluster analyses, an alternative model was devised in order to determine subgroups within the population. First, the vascular measures were transformed into z scores, one for each variable, calculated separately for males and females. The z scores were categorized into Vascular Status Scores (VSS) based on either median, tertiles and quintiles for the cIMT, PWV and AIX measurements, respectively. Each category was assigned a number, starting with 0, corresponding to the lowest category, adding 1 for each additional category. The range of scores was thus 0–3 for the median based score and 0–6 and 0–12 for the tertile and quintile based, respectively. For each individual, the assigned numbers of the three variables were added, forming the VSS_Median_, VSS_Tertile_ and VSS_Quintile_. A higher VSS indicates an unfavorable vascular structure and function.

Univariable regression models were performed to compare the VSS to the established risk factors. Multivariable regression models were then performed with the variables found to be significantly associated with the VSSs, to examine possible confounding factors and identify risk factors independently associated with an unfavorable vascular structure and function in a young population.

## Results

Baseline characteristics of the study population are shown in Table 1. Of the total population, 35 subjects (1.6% of the total population, 4 of which were females) had a systolic blood pressure above 140 mmHg during the first visit for examination. 13 of these (2 females) had a systolic blood pressure above 140 mmHg during the second measurement as well. None of the participants had a diastolic blood pressure above 90 mmHg. Blood pressure is included in the examination of PWV and AIX, and is an important predictor of increasing PWV in follow-up studies [35]. Significant differences in some of the lipid biomarker concentrations were seen between males and females (CHOL and Apo B/Apo A-1 ratio). Between men and estrogen users, all included lipid biomarkers differed significantly. Estrogen users had higher LDL and TG concentrations than non-estrogen users. The mean concentrations of the biomarkers of inflammation, CRP and orosomucoid, differed between estrogen users and non-estrogen users. As for the vascular structure and function measures, PWV differed between all groups. AIX differed between men and women, but no difference was seen based on contraceptive use. Sensitivity analysis with recalculation of Table 2, with the individuals who did not report the name of their contraceptive assigned to the non-estrogen group instead of the estrogen one, yielded similar results (Additional file 1: Table S1).

**Table 1:** Baseline characteristics of the studied population sample

	Males (*n* = 257)	Females, non-estrogen users (*n* = 428)	Females, estrogen users (*n* = 149)	*p* value M vs NEF	*p* value M vs EF	*p* value NEF vs EF
Age	22 ± 2.0	22 ± 2.0	22 ± 1.6	0.69	0.27	0.58
Body fat (%)	15 ± 5.6	28 ± 6.8	27 ± 5.9	< 0.001	< 0.001	0.42
LDL (mmol/L)	2.3 ± 0.69	2.2 ± 0.69	2.5 ± 0.79	0.36	< 0.001	< 0.001
HDL (mmol/L)	1.2 ± 0.28	1.4 ± 0.35	1.5 ± 0.41	< 0.001	< 0.001	0.81
CHOL (mmol/L)	4.0 ± 0,79	4.3 ± 0.77	4.4 ± 0.77	0.0034	< 0.001	0.0042
TG (mmol/L)	0.79 ± 0.35	0.75 ± 0.32	0.99 ± 0.39	0.25	< 0.001	< 0.001
Fasting serum insulin (mIE/L)	7.5 ± 3.7	8.1 ± 4.7	8.2 ± 4.4	0.32	0.56	0.99
Apo B (g/L)	0.77 ± 0.18	0.78 ± 0.18	0.83 ± 0.20	0.97	< 0.001	< 0.001
Apo A-1 (g/L)	1.4 ± 0.21	1.5 ± 0.27	1.7 ± 0.325	< 0.001	< 0.001	< 0.001
Apo B/Apo A-1 ratio	0.56 ± 0.14	0.51 ± 0.14	0.50 ± 0.15	< 0.001	0.032	0.75
CRP (mg/L)	1.3 ± 2.7	1.4 ± 2.8	4.1 ± 7.1	0.99	< 0.001	< 0.001
Orosomucoid (g/L)	0.72 ± 0.16	0.69 ± 0.17	0.60 ± 0.17	0.30	< 0.001	< 0.001
Systolic BP (mmHg)	122 ± 11	109 ± 9.0	112 ± 7.5	< 0.001	< 0.001	0.0034
Diastolic BP (mmHg)	64 ± 6.7	64 ± 6.0	65 ± 6.9	0.089	0.75	0.015
PWV (m/s)	5.6 ± 0.86	5.2 ± 0.69	5.3 ± 0.9	< 0.001	0.092	0.0045
AIX (%)	−8.4 ± 9.5	−5.0 ± 10	−4.7 ± 9.1	< 0.001	0.0019	0.99
cIMT (mm)	0.60 ± 0.073	0.49 ± 0.057	0.50 ± 0.055	0.48	0.18	0.61

Values are presented as mean ± SD (standard deviation). P value: comparison between groups by ANOVA with Tukey post hoc comparison. *M* males. *NEF* Non-estrogen using females. *EF* estrogen using females. *LDL* low-density lipoprotein. *HDL* high-density lipoprotein. *TG* triglycerides. *CHOL* total cholesterol. *Apo B* apolipoprotein *B. Apo A-1* Apolipoprotein A-1. *CRP* C-reactive protein. *BP* Blood pressure. *PWV* pulse-wave velocity. *AIX* Augmentation index. *cIMT* carotid-intima media thickness

**Table 2:** Univariable analysis. The relationship between Vascular Status Scores and biomarkers of cardiovascular risk in univariable analyses

	VSS_Median_		VSS_Tertile_		VSS_Quintile_	
	β _Median_ (95% CI)	*p*	β _Tertile_ (95% CI)	*p*	β _Quintile_ (95% CI)	*p*
LDL	0.10 (0.045; 0.16)	< 0.001	0.12 (0.024; 0.22)	0.014	0.25 (0.073; 0.42)	0.0054
HDL	−0.011 (− 0.070; 0.047)	0.71	−0.013 (− 0.11; 0.86)	0.80	0.008 (− 0.17; 0.18)	0.93
TG	0.014 (−0.044; 0.073)	0.63	0.089 (−0.0091; 0.19)	0.075	0.13 (−0.046; 0.30)	0.15
CHOL	0.97 (0.39; 0.16)	0.0011	0.12 (0.022; 0.22)	0.017	0.23 (0.061; 0.41)	0.0082
ApoB/ApoA-1 ratio	0.077 (0.019; 0.14)	0.010	0.068 (−0.031; 0.17)	0.18	0.14 (−0.032; 0.32)	0.11
Insulin	0.050 (−0.0091; 0.11)	0.097	0.11 (0.10; 0.21)	0.031	0.18 (0.005; 0.35)	0.044
Glucose	0.061 (0.020; 0.12)	0.043	0.10 (0.0021; 0.20)	0.045	0.17 (−0.005; 0.34)	0.056
Body fat percentage	0.10 (0.45; 0.16)	< 0.001	0.20 (0.10; 0.30)	< 0.001	0.36 (0.19; 0.54)	< 0.001
CRP	0.063 (0.0039; 0.12)	0.037	0.17 (0.075; 0.27)	< 0.001	0.31 (0.13; 0.48)	< 0.001
Orosomucoid	0.067 (0.0085; 0.13)	0.025	0.10 (0.0044; 0.20)	0.041	0.16 (−0.016; 0.33)	0.076
Estrogen contraceptive use (yes/no)	0.0074 (−0.16; 0.17)	0.93	0.13 (−0.15; 0.40)	0.36	0.30 (−0.19; 0.78)	0.23

*β* β coefficient. *CI* confidence interval. The variables were z score transformed before regression analysis. Abbreviations: see Table 1

**Fig. 1:**
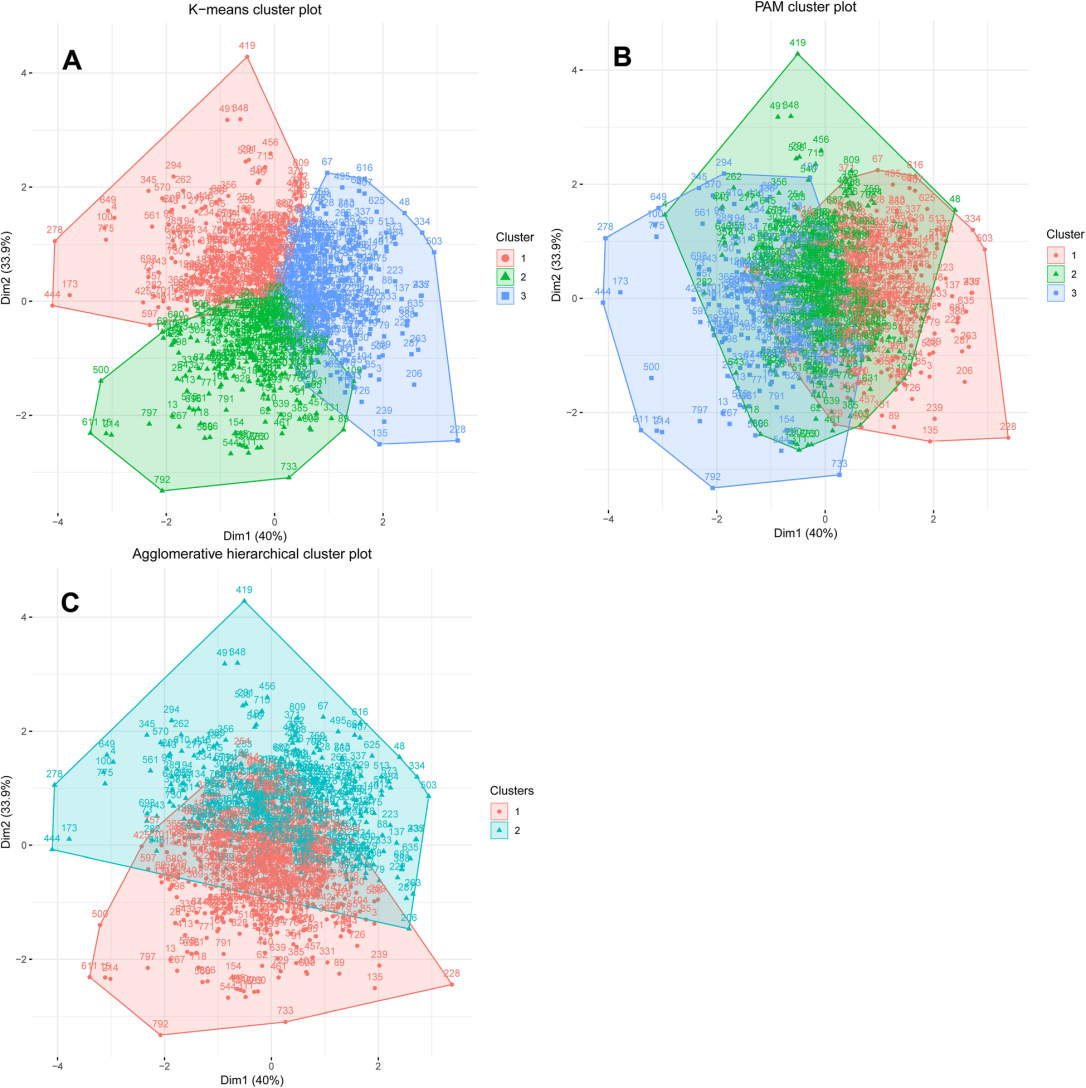
Clusters indicated by the first two principle components in the k-means (**a**), PAM (**b**) and AGNES (**c**) cluster analyses

**Fig. 2:**
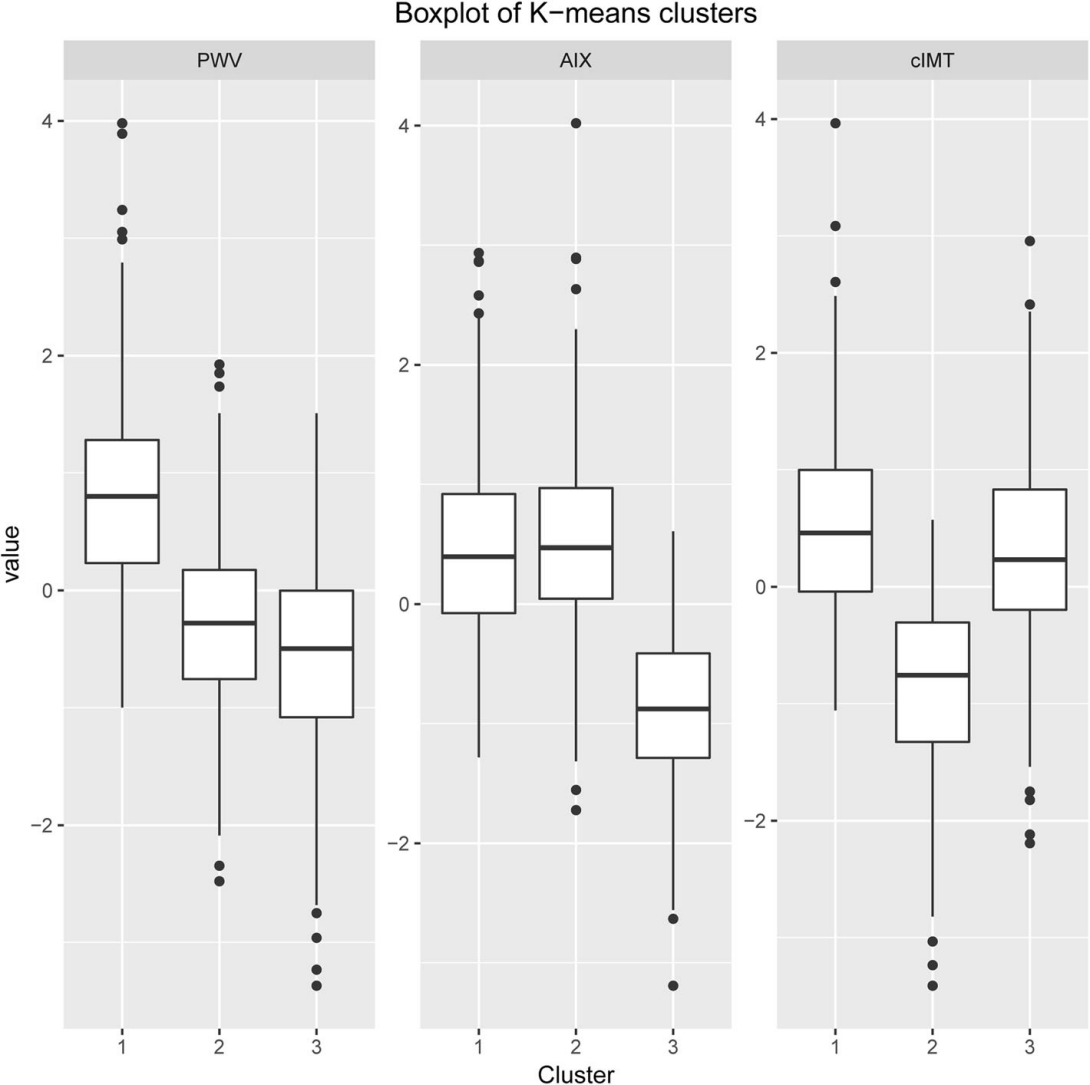
Boxplot visualization of the mean PWV, AIX and cIMT for each of the three clusters, as obtained by the k-means cluster analysis. The vascular variables are z-score transformed

The three measures of vascular structure and function, cIMT, PWV and AIX, were entered into cluster analyses using the different cluster analysis algorithms, AGNES, PAM and k-means clustering. None of these clustering algorithms yielded distinct clusters, corresponding to groups of individuals with a vascular structure and function distinctly associated with a higher or lower risk of CVD. A substantial overlap was seen between the formed clusters (Fig. 1). The k-means algorithm, when restricted to 3 clusters, fared the best (Fig. 1a); however, when plotting the mean values of the respective Z-scores for the three vascular structure and function measures (PWV, cIMT, AIX) in the three k-means clusters, it showed that the clusters did not manage to unequivocally separate subjects with the best vs. the poorest vascular health as defined by all three measures, i.e. low-low-low vs. high-high-high (Fig. 2).

We therefore tried an alternative approach, constructing a vascular status score (VSS; see Methods). The relationship between the VSS calculations with the established markers of cardiovascular risk was analyzed by univariable regression analyses, using the VSSs as the dependent variable, in separate analyses. As seen in Table 2, serum lipid biomarkers related to the LDL particles, LDL, CHOL and ApoB/ApoA-1 ratio, as well as metabolic biomarkers insulin and glucose were significantly associated with most of the VSS calculations. Lipid biomarkers HDL and TG, however, were not. Inflammatory biomarkers CRP and orosomucoid were significantly associated with all three VSSs, with only one exception (orosomucoid and VSS_Quintile_). In women, estrogen contraceptive use did was not significantly associated with either of the VSSs. A recalculation after reclassification of the individuals who did not report the name of their contraceptives gave rise to the same result (data not shown). In general, the analyses yielded similar results and *p* values, irrespective of the type of VSS used. The VSS_Quintile_ was chosen for further multivariable analysis. To examine possible independent associations between the VSS and the biomarker variables, the variables with a p value lower than 0.1 in the univariable analyses (Table 2) were included in the multivariable analysis (Table 3). Among these, the serum lipid biomarkers LDL and CHOL, and metabolic biomarkers insulin and glucose did not display an independently significant association with VSS_Quintile._ Despite a probably high collinearity among some of the biomarkers entered into the model, body fat percentage and inflammatory biomarker CRP remained significantly associated with VSS_Quintile_. The *R*^2^ of the final model was low: 0.027. As a sensitivity analysis, the same statistical analyses as in Table 2 and Table 3 were repeated excluding the 13 subjects with elevated systolic blood pressure at both test occasions. The results were similar in the sensitivity analysis with β coefficients and significances being in accordance with the original analyses (Additional file 1: Tables S2 and S3).

**Table 3:** Associations between VSS_Quintile_ and biomarkers of cardiovascular risk in multivariable regression analysis

	β (95% CI)	*p*
LDL	0.062 (−0.24; 0.36)	0.69
CHOL	0.14 (−0.16; 0.43)	0.34
Insulin	−0.037 (− 0.24; 0.17)	0.72
Glucose	0.14 (−0.51; 0.32)	0.16
Body fat percentage	0.27 (0.73; 0.46)	0.0068
CRP	0.22 (0.026; 0.41)	0.029
Orosomucoid	−0.004 (−0.20; 0.19)	0.97

Abbreviations: see Table 1

Of the variables in the univariate analysis (Table 2), only those with a *p* value < 0.1 in univariable analyses were entered into the equation

## Discussion

Theoretical advances in the understanding of how risk factors for atherosclerosis start operating long before any clinical diagnoses have become apparent are increasingly recognized [36, 37], and highlight the necessity to start intervention efforts in individuals at risk as early as possible. The vascular health at the young age studied in the LBA cohort (18–26 yr) is likely to be only subtly altered even in the most vulnerable subgroup(s) as the study population was selected for being healthy, excluding individuals diagnosed with any disease that could have an effect on vascular measurements or biomarkers. In a healthy population with a low pre-test risk, the choice of risk stratification modality would ideally be made based on its sensitivity of detection of minor disadvantageous changes in an early, asymptomatic stage, as well as whether the modality chosen confers any additional risk to the subjects, i.e. only non-invasive tests are warranted [38]. Risk assessment modalities recommended in the management of stable CVD, such as nuclear imaging or computer tomography based examination [39] may not be validated or feasible as tools for a population screening. cIMT is well-established as a surrogate measurement of risk. It is described to increase 0.6 μm/year and to be associated with increased risk in the longitudinal Cardiovascular Risk in Young Finns Study [40]. PWV, AIX and cIMT, the modalities of choice in this study have been shown to be associated with risk factors for future CVD in children. The strongest associations were found in children with more obvious risk, such as obesity and non-alcoholic fatty liver disease [41–43]. In our population, albeit older, but selected for being healthy the associations found between VSS and biomarkers of CVD risk are likely to explain a rather low percentage of the total variance of VSSs in our sample. It is likely that the benefit of the VSS calculation would increase with increasing age of the subjects as more and more would have started to develop adverse changes in both vascular structure and function measurements. Nonetheless, any demonstrable associations suggest that endeavors towards combating such risk factors have a prioritized role in the prevention or slowing of atherosclerotic progression in young individuals. In this study, we found that the adverse consequences of an increased body fat percentage and systemic inflammation independently correlate with arterial physiology, and is detectable as a worsening of vascular measurements in a process that begins already in early adulthood. Among females, there was a significant difference in mean PWV between estrogen users and non-estrogen users in the population (Table 1). However, no relationship between contraceptive use and vascular structure and function, as estimated by the VSS, was found in the regression models (Table 2). The possible effect of estrogen on the endothelial function is likely subtle and could be thought to affect arterial stiffness measurements, such as PWV, before an effect of vascular remodeling, such as increased cIMT, is seen. Previous investigations on the effect of exogenous estrogen on vascular health have mainly examined postmenopausal women and have given contradictive results, with some studies reporting beneficial effects and others not [16, 17, 44, 45]. To our knowledge, only three studies have examined arterial stiffness in relation to estrogen contraceptive use. As was the case in our population (Table 1), one of the studies found a higher PWV among estrogen contraceptive users [46], while two studies [47, 48] found no such difference. In summary, any possible estrogen effect on vascular status measures is likely subtle in premenopausal women.

Cardiovascular risk profiling based on blood biomarkers is often improved when based on more variables [38, 49]. Similarly, the limitation of individual vascular status measures suggests that using more than one marker yields a more reliable appraisal of an individual’s vascular status and risk [15, 50, 51]. The cluster analyses are pattern oriented, established on assumptions of the existence of subjacent structures in the data set and based on grouping of individuals [52], while the vascular status score (VSS) introduced here determines subgroup profiles based on the sum of scores of the included variables. The individuals who formed the extreme groups, corresponding to the subjects having the best or worst vascular structure and function according to the VSS_Quintile_, were not identified as constituting an unfavorable subgroup by any of the cluster analyses. A variable oriented approach (VSS) thus performed better in singling them out as belonging to an extreme category with respect to their vascular status.

In univariable analyses, LDL, TG, CHOL, fasting insulin, body fat percentage and CRP were significantly associated with VSS_Quintile_, but not HDL, Apo B/Apo A-1 ratio, glucose, orosomucoid or estrogen contraceptive use in women. In multivariable analyses, only body fat percentage and CRP remained independently significant predictors of VSS_Quintile_ (Table 3). CRP is well established as a risk factor [53]. In children as young as 8–9 of age, CRP concentration is associated with higher arterial stiffness [54]. The association between CRP and body fat percentage and vascular measurements remained significant upon sensitivity analysis, excluding the hypertensive subjects, which emphasizes the robustness of the association and the importance of inflammation and adiposity in vascular health in this population. The *R*^*2*^ of the final model was low, as expected in a cohort of young subjects selected for health by excluding smokers, diabetes, and other chronic diseases. Based on Natural Randomisation studies and many traditional CVD risk studies [55, 56] there is no doubt that lipid status (especially LDL cholesterol) and metabolic syndrome with peripheral insulin resistance, play important roles for CVD events in older age in subjects with more pronounced atherosclerosis in place. The fact that these biomarkers were not found to be significant in this study suggests that the earliest drivers of the pathophysiological process are adiposity and associated low-grade inflammation, as seen in the multivariable analysis, whereas lipid accumulation and macro- and microvasculopathy develop at a later stage. The observed variation in PWV and AIX measurements and their relation with biomarkers of risk in the population are in accordance with the idea that pathophysiologically, endothelial dysfunction comes early in the atherosclerotic progression. Dysfunctionality such as paradoxical vasoconstriction is seen in mild CVD [57]. Similarly, the inflammatory component of the pathophysiology of atherosclerosis is thought to come early, in the form of an interplay between the immunologically active endothelium and other tissues, such as the liver, responding to proinflammatory signaling instigated by endothelial receptor interaction with various substances [58]. This proinflammatory activity is probably detectable at an early stage, by measurement of CRP, whereas the resulting buildup such as smooth muscle cell proliferation and leucocyte recruitment require a longer duration of inflammatory load before being detectable and making an impact on the vascular function and structure [59, 60]. While well established as an important risk marker and mediator [55], serum lipid concentration seemed to have less of an impact at the early stage in the LBA population of young, healthy subjects.

The methodologies used in this study were chosen for being feasible and non-invasive, but it is possible that other modalities would have been more sensitive. The cross-sectional design of this study does not permit inferences of causality in the associations found between an unfavorable vascular status, and an increased body fat percentage and CRP. However, a wealth of evidence from the 1980’s and onwards has indicated inflammation as a main mediator in the progressive endothelial dysfunction that is seen in atherosclerosis [14, 61]. Similarly, childhood obesity is linked to an increased cardiovascular risk in adulthood compared to individuals whose obesity started in adulthood [62, 63]. Inflammation has been suggested as the key regulatory process linking multiple risk factors, including obesity, to the onset of atherosclerosis [61].

## Conclusions

The cluster analyses of vascular structure and function measures yielded unsatisfactory results (i.e. poor discrimination between subjects having a vascular structure and function profile associated with an unequivocally high or low CVD risk) in this study of young healthy individuals, probably due to the close collinearity of these measures. Therefore, we devised a variable oriented approach, by scoring the measures of vascular structure and function to compose VSS as a surrogate endpoint in the context of the LBA population. The serum concentration of C-reactive protein and the body fat percentage were the only independently significant predictors of VSS_Quintile_ in this cohort. These findings highlight the important role of chronic low-grade inflammation in the vascular health in young adults, and underlines the importance of counteracting adiposity already in young adulthood in preventing premature onset of preclinical atherosclerosis. Further studies are warranted to establish relevant cut-off values for clinical use in the preventive work in low risk groups.

## Supplementary information

**Additional file 1.** Sensitivity analysis recalculation of the main Tables 2 and 3 from the manuscript, by reassignment of a portion of the subjects based on their blood pressure and estrogen contraceptive use.

## Data Availability

The datasets generated and/or analysed during the current study are not publicly available due to research subject confidentiality but are available in a de-identified form from the corresponding author on reasonable request and with permission of the PI of the study.

## Abbreviations

AGNES: Agglomerative nesting hierarchical cluster analysis
AIX: Augmentation index
Apo A-1: Apolipoprotein A-1
Apo B: Apolipoprotein B
CHOL: Total cholesterol
cIMT: Carotid-intima media thickness
CRP: C-reactive protein
CVD: Cardiovascular disease
HDL: High-density lipoprotein
LDL: Low-density lipoprotein
PAM: Partitioning around medoids cluster analysis
PWV: Pulse-wave velocity
TG: Trigycerides
VSS: Vascular status score

## References

[1] Messner B, Bernhard D (2014). Smoking and cardiovascular disease: mechanisms of endothelial dysfunction and early atherogenesis. Arterioscler Thromb Vasc Biol.

[2] Bentzon JF, Otsuka F, Virmani R, Falk E (2014). Mechanisms of plaque formation and rupture. Circ Res.

[3] Berenson GS, Srinivasan SR, Nicklas TA (1998). Atherosclerosis: a nutritional disease of childhood. Am J Cardiol.

[4] McMahan CA, Gidding SS, Malcom GT, Tracy RE, Strong JP, McGill HC (2006). Pathobiological determinants of atherosclerosis in youth risk scores are associated with early and advanced atherosclerosis. Pediatrics..

[5] Jalowiec DA, Hill JA (1989). Myocardial infarction in the young and in women. Cardiovasc Clin.

[6] Klein LW, Nathan S (2003). Coronary artery disease in young adults. J Am Coll Cardiol.

[7] Hallenbeck JM, Hansson GK, Becker KJ (2005). Immunology of ischemic vascular disease: plaque to attack. Trends Immunol.

[8] Celik O, Ozturk D, Akin F, Satilmis S, Yalcin AA, Erturk M (2015). Evaluation of lipoprotein-associated phosholipase A2 and plaque burden/composition in young adults. Coron Artery Dis.

[9] Rajala U, Laakso M, Paivansalo M, Pelkonen O, Suramo I, Keinanen-Kiukaanniemi S (2002). Low insulin sensitivity measured by both quantitative insulin sensitivity check index and homeostasis model assessment method as a risk factor of increased intima-media thickness of the carotid artery. J Clin Endocrinol Metab.

[10] Oikonen M, Laitinen TT, Magnussen CG, Steinberger J, Sinaiko AR, Dwyer T (2013). Ideal cardiovascular health in young adult populations from the United States, Finland, and Australia and its association with cIMT: the international childhood cardiovascular cohort consortium. J Am Heart Assoc.

[11] Rosenbaum D, Giral P, Chapman J, Rached FH, Kahn JF, Bruckert E (2013). Radial augmentation index is a surrogate marker of atherosclerotic burden in a primary prevention cohort. Atherosclerosis..

[12] Bauer M, Caviezel S, Teynor A, Erbel R, Mahabadi AA, Schmidt-Trucksass A (2012). Carotid intima-media thickness as a biomarker of subclinical atherosclerosis. Swiss Med Wkly.

[13] Pereira T, Maldonado J, Polonia J, Silva JA, Morais J, Rodrigues T (2014). Aortic pulse wave velocity and HeartSCORE: improving cardiovascular risk stratification. A sub-analysis of the EDIVA (Estudo de DIstensibilidade VAscular) project. Blood Press.

[14] Maloberti A, Vallerio P, Triglione N, Occhi L, Panzeri F, Bassi I, Pansera F, Piccinelli E, Peretti A, Garatti L, Palazzini M, Sun J, Grasso E, Giannattasio C (2019). Vascular aging and disease of the large vessels: role of inflammation. High Blood Press Cardiovasc Prev.

[15] Bruno RM, Bianchini E, Faita F, Taddei S, Ghiadoni L (2014). Intima media thickness, pulse wave velocity, and flow mediated dilation. Cardiovasc Ultrasound.

[16] Tentolouris N, Christodoulakos G, Lambrinoudaki I, Mandalaki E, Panoulis C, Maridaki C (2005). Effect of hormone therapy on the elastic properties of the arteries in healthy postmenopausal women. J Endocrinol Investig.

[17] Teede HJ, Liang YL, Kotsopoulos D, Zoungas S, Cravent R, McGrath BP (2001). A placebo-controlled trial of long-term oral combined continuous hormone replacement therapy in postmenopausal women: effects on arterial compliance and endothelial function. Clin Endocrinol.

[18] Angerer P, Stork S, Kothny W, Schmitt P, von Schacky C (2001). Effect of oral postmenopausal hormone replacement on progression of atherosclerosis : a randomized, controlled trial. Arterioscler Thromb Vasc Biol.

[19] Hodis HN, Mack WJ, Lobo RA, Shoupe D, Sevanian A, Mahrer PR (2001). Estrogen in the prevention of atherosclerosis. A randomized, double-blind, placebo-controlled trial. Ann Intern Med.

[20] Laurell CB, Kullander S, Thorell J (1968). Effect of administration of a combined estrogen-progestin contraceptive on the level of individual plasma proteins. Scand J Clin Lab Invest.

[21] Laurell CB, Rannevik G (1979). A comparison of plasma protein changes induced by danazol, pregnancy, and estrogens. J Clin Endocrinol Metab.

[22] Pettersson-Pablo P, Nilsson TK, Breimer LH, Hurtig-Wennlof A. Body fat percentage is more strongly associated with biomarkers of low-grade inflammation than traditional cardiometabolic risk factors in healthy young adults - the lifestyle, biomarkers, and atherosclerosis study. Scand J Clin Lab Invest. 2019:1–6. 10.1080/00365513.2019.1576219 30767573

[23] Hofstetter H, Dusseldorp E, van Empelen P, Paulussen TW (2014). A primer on the use of cluster analysis or factor analysis to assess co-occurrence of risk behaviors. Prev Med.

[24] Lind L (2014). Flow-mediated vasodilation over five years in the general elderly population and its relation to cardiovascular risk factors. Atherosclerosis..

[25] Rudolf J, Lewandrowski KB (2014). Cholesterol, lipoproteins, high-sensitivity c-reactive protein, and other risk factors for atherosclerosis. Clin Lab Med.

[26] Ren L, Cai J, Liang J, Li W, Sun Z (2015). Impact of cardiovascular risk factors on carotid intima-media thickness and degree of severity: a cross-sectional study. PLoS One.

[27] Taft C, Karlsson J, Sullivan M (2004). Performance of the Swedish SF-36 version 2.0. Qual Life Res.

[28] Läkemedelsverket (Swedish Medical Products Agency). Antikonception. 16 April 2014 [Cited 28 October 2019]. Avialable from: https://lakemedelsverket.se/antikonception.

[29] Fernstrom M, Fernberg U, Eliason G, Hurtig-Wennlof A (2017). Aerobic fitness is associated with low cardiovascular disease risk: the impact of lifestyle on early risk factors for atherosclerosis in young healthy Swedish individuals - the lifestyle, biomarker, and atherosclerosis study. Vasc Health Risk Manag.

[30] Touboul PJ, Hennerici MG, Meairs S, Adams H, Amarenco P, Bornstein N (2012). Mannheim carotid intima-media thickness and plaque consensus (2004-2006-2011). An update on behalf of the advisory board of the 3rd, 4th and 5th watching the risk symposia, at the 13th, 15th and 20th European stroke conferences, Mannheim, Germany, 2004, Brussels, Belgium, 2006, and Hamburg, Germany, 2011. Cerebrovasc Dis.

[31] Mackenzie IS, Wilkinson IB, Cockcroft JR (2002). Assessment of arterial stiffness in clinical practice. QJM..

[32] Fernberg U, Fernstrom M, Hurtig-Wennlof A (2017). Arterial stiffness is associated to cardiorespiratory fitness and body mass index in young Swedish adults: the lifestyle, biomarkers, and atherosclerosis study. Eur J Prev Cardiol.

[33] Laurent S, Cockcroft J, Van Bortel L, Boutouyrie P, Giannattasio C, Hayoz D (2006). Expert consensus document on arterial stiffness: methodological issues and clinical applications. Eur Heart J.

[34] Pauca AL, O'Rourke MF, Kon ND (2001). Prospective evaluation of a method for estimating ascending aortic pressure from the radial artery pressure waveform. Hypertension..

[35] Meani P, Maloberti A, Sormani P, Colombo G, Giupponi L, Stucchi M, Varrenti M, Vallerio P, Facchetti R, Grassi G, Mancia G, Giannattasio C (2018). Determinants of carotid-femoral pulse wave velocity progression in hypertensive patients over a 3.7 years follow-up. Blood Press.

[36] Ference BA, Yoo W, Alesh I, Mahajan N, Mirowska KK, Mewada A (2012). Effect of long-term exposure to lower low-density lipoprotein cholesterol beginning early in life on the risk of coronary heart disease: a Mendelian randomization analysis. J Am Coll Cardiol.

[37] Holmes MV, Asselbergs FW, Palmer TM, Drenos F, Lanktree MB, Nelson CP (2015). Mendelian randomization of blood lipids for coronary heart disease. Eur Heart J.

[38] Iyngkaran P, Noaman S, Chan W, Mahadavan G, Thomas MC, Rajendran S (2019). Non-invasive risk stratification for coronary artery disease: is it time for subclassifications?. Curr Cardiol Rep.

[39] Dancy L, O'Gallagher K, Milton P, Sado D (2018). New NICE guidelines for the management of stable angina. Br J Gen Pract.

[40] Raitakari Olli T., Juonala Markus, Kähönen Mika, Taittonen Leena, Laitinen Tomi, Mäki-Torkko Noora, Järvisalo Mikko J., Uhari Matti, Jokinen Eero, Rönnemaa Tapani, Åkerblom Hans K., Viikari Jorma S. A. (2003). Cardiovascular Risk Factors in Childhood and Carotid Artery Intima-Media Thickness in Adulthood. JAMA.

[41] Cote AT, Harris KC, Panagiotopoulos C, Sandor GG, Devlin AM (2013). Childhood obesity and cardiovascular dysfunction. J Am Coll Cardiol.

[42] Manco M, Bedogni G, Monti L, Morino G, Natali G, Nobili V (2010). Intima-media thickness and liver histology in obese children and adolescents with non-alcoholic fatty liver disease. Atherosclerosis..

[43] Manco M, Nobili V, Alisi A, Panera N, Handberg A (2017). Arterial stiffness, thickness and association to suitable novel markers of risk at the origin of cardiovascular disease in obese children. Int J Med Sci.

[44] Hodis HN, Mack WJ, Henderson VW, Shoupe D, Budoff MJ, Hwang-Levine J (2016). Vascular effects of early versus late postmenopausal treatment with estradiol. N Engl J Med.

[45] Naessen T, Rodriguez-Macias K (2006). Menopausal estrogen therapy counteracts normal aging effects on intima thickness, media thickness and intima/media ratio in carotid and femoral arteries. An investigation using noninvasive high-frequency ultrasound. Atherosclerosis..

[46] Hickson SS, Miles KL, McDonnell BJ (2011). Yasmin, Cockcroft JR, Wilkinson IB, et al. use of the oral contraceptive pill is associated with increased large artery stiffness in young women: the ENIGMA study. J Hypertens.

[47] Yu A, Giannone T, Scheffler P, Doonan RJ, Egiziano G, Gomez YH (2014). The effect of oral contraceptive pills and the natural menstrual cYCLe on arterial stiffness and hemodynamICs (CYCLIC). J Hypertens.

[48] Priest SE, Shenouda N, MacDonald MJ (2018). Effect of sex, menstrual cycle phase, and monophasic oral contraceptive pill use on local and central arterial stiffness in young adults. Am J Physiol Heart Circ Physiol.

[49] Price AH, Weir CJ, Welsh P, McLachlan S, Strachan MWJ, Sattar N (2017). Comparison of non-traditional biomarkers, and combinations of biomarkers, for vascular risk prediction in people with type 2 diabetes: the Edinburgh type 2 diabetes study. Atherosclerosis..

[50] McIntosh MW, Pepe MS (2002). Combining several screening tests: optimality of the risk score. Biometrics..

[51] Liu C, Liu A, Halabi S (2011). A min-max combination of biomarkers to improve diagnostic accuracy. Stat Med.

[52] Hemming K, Eldridge S, Forbes G, Weijer C, Taljaard M (2017). How to design efficient cluster randomised trials. BMJ..

[53] Libby P, Ridker PM, Hansson GK (2011). Progress and challenges in translating the biology of atherosclerosis. Nature..

[54] Correia-Costa A, Correia-Costa L, Caldas Afonso A, Schaefer F, Guerra A, Moura C, Mota C, Barros H, Areias JC, Azevedo A (2016). Determinants of carotid-femoral pulse wave velocity in prepubertal children. Int J Cardiol.

[55] Ference BA, Ray KK, Catapano AL, Ference TB, Burgess S, Neff DR (2019). Mendelian randomization study of ACLY and cardiovascular disease. N Engl J Med.

[56] Paneni F, Costantino S, Cosentino F (2014). Insulin resistance, diabetes, and cardiovascular risk. Curr Atheroscler Rep.

[57] Ludmer PL, Selwyn AP, Shook TL, Wayne RR, Mudge GH, Alexander RW, Ganz P (1986). Paradoxical vasoconstriction induced by acetylcholine in atherosclerotic coronary arteries. N Engl J Med.

[58] Lundberg AM, Hansson GK (2010). Innate immune signals in atherosclerosis. Clin Immunol.

[59] Warner SJ, Auger KR, Libby P (1987). Interleukin 1 induces interleukin 1. II. Recombinant human interleukin 1 induces interleukin 1 production by adult human vascular endothelial cells. J Immunol.

[60] Cybulsky MI, Lichtman AH, Hajra L, Iiyama K (1999). Leukocyte adhesion molecules in atherogenesis. Clin Chim Acta.

[61] Rocha VZ, Libby P (2009). Obesity, inflammation, and atherosclerosis. Nat Rev Cardiol.

[62] Herouvi D, Karanasios E, Karayianni C, Karavanaki K (2013). Cardiovascular disease in childhood: the role of obesity. Eur J Pediatr.

[63] Keustermans GC, Kofink D, Eikendal A, de Jager W, Meerding J, Nuboer R (2017). Monocyte gene expression in childhood obesity is associated with obesity and complexity of atherosclerosis in adults. Sci Rep.

