# Supplementary material for: Body fat percentage and CRP correlates with a composite score of vascular risk markers in healthy, young adults - The Lifestyle, Biomarkers, and Atherosclerosis (LBA) study
Source: BMC Cardiovasc Disord. 2020 Feb 11;20:77. doi: 10.1186/s12872-020-01376-6 (PMC7014751; doi:10.1186/s12872-020-01376-6)
Supplement: Supplementary file 1 — Additional file 1. Sensitivity analysis recalculation of the main Tables 2 and 3 from the manuscript, by reassignment of a portion of the subjects based on their blood pressure and estrogen contraceptive use. [file 12872_2020_1376_MOESM1_ESM.docx]

**SUPPLEMENTARY TABLES**

**Supplementary table 1**

**Main Table 2 recalculated upon reclassification of the females who did not report the name of their contraceptive. Univariable analysis. The relationship between Vascular Status Scores and biomarkers of cardiovascular risk in univariable analyses.**

|  | VSS_Median_ | | |  | VSS_Tertile_ | | |  | | VSS_Quintile_ | | | |
| --- | --- | --- | --- | --- | --- | --- | --- | --- | --- | --- | --- | --- | --- |
|  | β _Median_ (95% CI) |  | *p* |  | β _Tertile_ (95% CI) |  | *p* |  | β _Quintile_ (95% CI) | |  | *p* |  |
| LDL | 0.10 (0.045; 0.16) |  | <0.001 |  | 0.12 (0.024; 0.22) |  | 0.014 |  | 0.25 (0.073; 0.42) | |  | 0.0054 |  |
|  |  |  |  |  |  |  |  |  |  | |  |  |  |
| HDL | -0.011 (-0.070 ; 0.047) |  | 0.71 |  | -0.013 (-0.11; 0.86) |  | 0.80 |  | 0.008 (-0.17; 0.18) | |  | 0.93 |  |
|  |  |  |  |  |  |  |  |  |  | |  |  |  |
| TG | 0.014 (-0.044; 0.073) |  | 0.63 |  | 0.089 (-0.0091; 0.19) |  | 0.075 |  | 0.13 (-0.046; 0.30) | |  | 0.15 |  |
|  |  |  |  |  |  |  |  |  |  | |  |  |  |
| CHOL | 0.97 (0.39; 0.16) |  | 0.0011 |  | 0.12 (0.022; 0.22) |  | 0.017 |  | 0.23 (0.061; 0.41) | |  | 0.0082 |  |
|  |  |  |  |  |  |  |  |  |  | |  |  |  |
| ApoB/ApoA-1 ratio | 0.077 (0.019; 0.14) |  | 0.010 |  | 0.068 (-0.031; 0.17) |  | 0.18 |  | 0.14 (-0.032; 0.32) | |  | 0.11 |  |
|  |  |  |  |  |  |  |  |  |  | |  |  |  |
| Insulin | 0.050 (-0.0091; 0.11) |  | 0.097 |  | 0.11 (0.10; 0.21) |  | 0.031 |  | 0.18 (0.005; 0.35) | |  | 0.044 |  |
|  |  |  |  |  |  |  |  |  |  | |  |  |  |
| Glucose | 0.061 (0.020; 0.12) |  | 0.043 |  | 0.10 (0.0021; 0.20) |  | 0.045 |  | 0.17 (-0.005; 0.34) | |  | 0.056 |  |
|  |  |  |  |  |  |  |  |  |  | |  |  |  |
| Body fat percentage | 0.10 (0.45; 0.16) |  | <0.001 |  | 0.20 (0.10; 0.30) |  | <0.001 |  | 0.36 (0.19; 0.54) | |  | <0.001 |  |
|  |  |  |  |  |  |  |  |  |  | |  |  |  |
| CRP | 0.063 (0.0039; 0.12) |  | 0.037 |  | 0.17 (0.075; 0.27) |  | <0.001 |  | 0.31 (0.13; 0.48) | |  | <0.001 |  |
|  |  |  |  |  |  |  |  |  |  | |  |  |  |
| Orosomucoid | 0.067 (0.0085; 0.13) |  | 0.025 |  | 0.10 (0.0044; 0.20) |  | 0.041 |  | 0.16 (-0.016; 0.33) | |  | 0.076 |  |
|  |  |  |  |  |  |  |  |  |  | |  |  |  |
| Estrogen contraceptive use (yes/no) | -0.0092 (-0.23; 0.22) |  | 0.94 |  | 0.12 (-0.25; 0.49) |  | 0.53 |  | 0.18 (-0.47; 0.82) | |  | 0.59 |  |

β = β coefficient. CI = confidence interval. The variables were z score transformed before regression analysis. Abbreviations: see Table 1.

**Supplementary table 2**

**Main Table 2 recalculated excluding hypertensive subjects. Univariable analysis. The relationship between Vascular Status Scores and biomarkers of cardiovascular risk in univariable analyses.**

|  | VSS_Median_ | | |  | VSS_Tertile_ | | |  | | VSS_Quintile_ | | | |
| --- | --- | --- | --- | --- | --- | --- | --- | --- | --- | --- | --- | --- | --- |
|  | β _Median_ |  | *p* |  | β _Tertile_ |  | *p* |  | β _Quintile_ (95% CI) | |  | *p* |  |
| LDL | 0.10 |  | 0.0011 |  | 0.072 |  | 0.039 |  | 0.085 | |  | 0.015 |  |
|  |  |  |  |  |  |  |  |  |  | |  |  |  |
| HDL | -0.0021 |  | 0.95 |  | 0.0011 |  | 0.97 |  | 0.016 | |  | 0.65 |  |
|  |  |  |  |  |  |  |  |  |  | |  |  |  |
| TG | 0.007 |  | 0.83 |  | 0.053 |  | 0.13 |  | 0.040 | |  | 0.25 |  |
|  |  |  |  |  |  |  |  |  |  | |  |  |  |
| CHOL | 0.12 |  | 0.0021 |  | 0.073 |  | 0.036 |  | 0.084 | |  | 0.016 |  |
|  |  |  |  |  |  |  |  |  |  | |  |  |  |
| ApoB/ApoA-1 ratio | 0.076 |  | 0.029 |  | 0.031 |  | 0.37 |  | 0.040 | |  | 0.26 |  |
|  |  |  |  |  |  |  |  |  |  | |  |  |  |
| Insulin | 0.043 |  | 0.22 |  | 0.057 |  | 0.10 |  | 0.050 | |  | 0.16 |  |
|  |  |  |  |  |  |  |  |  |  | |  |  |  |
| Glucose | 0.064 |  | 0.066 |  | 0.061 |  | 0.079 |  | 0.059 | |  | 0.090 |  |
|  |  |  |  |  |  |  |  |  |  | |  |  |  |
| Body fat percentage | 0.11 |  | <0.001 |  | 0.13 |  | <0.001 |  | 0.13 | |  | <0.001 |  |
|  |  |  |  |  |  |  |  |  |  | |  |  |  |
| CRP | 0.061 |  | 0.081 |  | 0.11 |  | <0.001 |  | 0.11 | |  | 0.0020 |  |
|  |  |  |  |  |  |  |  |  |  | |  |  |  |
| Orosomucoid | 0.068 |  | 0.052 |  | 0.062 |  | 0.076 |  | 0.050 | |  | 0.15 |  |
|  |  |  |  |  |  |  |  |  |  | |  |  |  |
| Contraceptive use (females only) | 0.055 |  | 0.46 |  | 0.10 |  | 0.42 |  | 0.23 | |  | 0.29 |  |

β = β coefficient. CI = confidence interval. The variables were z score transformed before regression analysis. LDL = low-density lipoprotein. HDL = high-density lipoprotein. TG = triglycerides. CHOL = total cholesterol. Apo B/Apo A-1 ratio = apolipoprotein B/Apolipoprotein A-1 ratio. CRP = C-reactive protein.

**Supplementary table 3**

**Main Table 3 recalculated excluding hypertensive subjects. Associations between VSSQuintile and biomarkers of cardiovascular risk in multivariable regression analysis.**

**Variables with a p value < 0.1 in univariable analyses were entered into the equation.**

|  |  |  | | |
| --- | --- | --- | --- | --- |
|  |  | β (95% CI) |  | *p* |
|  |  |  |  |  |
| LDL |  | 0.037 (-0.26; 0.34) |  | 0.81 |
|  |  |  |  |  |
| CHOL |  | 0.14 (-0.16; 0.44) |  | 0.35 |
|  |  |  |  |  |
| Insulin |  | -0.070 (-0.28; 0.14) |  | 0.51 |
|  |  |  |  |  |
| Glucose |  | 0.14 (-0.53; 0.32) |  | 0.16 |
|  |  |  |  |  |
| Body fat percentage |  | 0.26 (0.063; 0.46) |  | 0.010 |
|  |  |  |  |  |
| CRP |  | 0.20 (0.015; 0.40) |  | 0.035 |
|  |  |  |  |  |
| Orosomucoid |  | -0.012 (-0.20; 0.18) |  | 0.91 |

β = β coefficient. CI = confidence interval. LDL = low-density lipoprotein. TG = triglycerides. CHOL = total cholesterol. Apo B/Apo A-1 ratio = apolipoprotein B/Apolipoprotein A-1 ratio. CRP = C-reactive protein.
